# Highly-Exposed HIV-1 seronegative Female Commercial Sex Workers sustain in their genital mucosa increased frequencies of tolerogenic myeloid and regulatory T-cells

**DOI:** 10.1038/srep43857

**Published:** 2017-03-06

**Authors:** V. Thibodeau, L. Fourcade, A.-C. Labbé, M. Alary, F. Guédou, J. Poudrier, M. Roger

**Affiliations:** 1 Laboratoire d’immunogénétique, Centre de Recherche du Centre Hospitalier de l’Université de Montréal (CRCHUM), Montréal, Canada; 2 Département de Microbiologie, Infectiologie et Immunologie de l’Université de Montréal, Montréal, Canada; 3 Centre de recherche du CHU de Québec, Québec, Canada; 4 Département de médecine sociale et préventive, Université Laval, Québec, Canada; 5 Dispensaire des IST, Cotonou, Benin.

## Abstract

We and others have shown that HIV-1 highly-exposed seronegative (HESN) female commercial sex workers (CSWs) maintain low genital inflammatory conditions to prevent HIV infection. HIV-1 interacts with toll-like receptors (TLR)-7/8 to induce interferon (IFN)-α, an important antiviral and immunomodulatory cytokine, which act together with interleukin (IL)-10, human leukocyte antigen (HLA)-G and immunoglobulin-like transcript (ILT)-4 to initiate a “tolerogenic/regulatory” anti-inflammatory loop. In view of further unravelling elements associated with natural immunity to HIV-1, we have characterised TLR-7, IFN-α, IL-10, HLA-G and ILT-4 expression profiles in the genital tract of female CSWs and HIV-1-uninfected non-CSWs from Benin. Endocervical myeloid HLA-DR^+^ cells from HESN CSWs expressed higher levels of IFN-α, TLR-7, IL-10 and HLA-G than those from both HIV-1-infected CSWs and HIV-1-uninfected non-CSWs. Further characterization of the endocervical myeloid HLA-DR^+^ cells in HESN CSWs revealed a population of “tolerogenic” CD103^+^ CD14^+^ CD11c^+^ myeloid cells expressing high levels of IFN-α and IL-10. Concomitantly, HESN CSWs had higher frequencies of endocervical regulatory CD4^+^ T-cells when compared to those from the two other groups of women. These novel findings provide strong evidence to support the implication of tolerogenic myeloid cells expressing high levels of antiviral molecules in shaping the genital mucosal immune response to prevent HIV infection.

In 2015, an estimated 36.7 million people were living with HIV/AIDS worldwide. Most HIV-1 infections are acquired through heterosexual intercourse, and in Africa, 60% of new HIV-1 infections affect women^1^. Vaccines and microbicides hold promise for preventing the acquisition of HIV-1, but the success of designing such agents needs a better understanding of the mechanisms of transmission and HIV-specific immune responses at the initial site of infection.

The female genital tract (FGT) constitutes a main portal of entry for HIV-1, and plays a critical role in protecting the host against pathogens while maintaining a tolerance to a commensal flora^2^,^3^. To this end, the FGT is provided with an array of protective mechanisms from the innate and adaptive arms of the immune system to maintain a delicate balance between protection and tolerance^4^. FGT immunity is also tightly regulated by a hormonal/inflammatory process throughout the menstrual cycle, having to deal with the pressure of procreation and microbial control^5^,^6^. The innate immune compartment of the FGT involves genital epithelial cells (GEC), dendritic cells (DC), Langherans cells (LC), macrophages, natural killer (NK) cells and neutrophils, which confer protection through the production of antimicrobial agents, chemokines and cytokines^7^,^8^. GECs, which form an uninterrupted barrier between the lumen and underlying cells, have been shown to express toll-like receptors (TLRs) 1 to 9, indicating the potential to respond to a wide range of microbes/pathogens^7^,^9^. It has been shown that mucosal epithelial cells (genital and intestinal) respond directly to envelope glycoproteins of HIV-1 by upregulating inflammatory cytokines^10^. Given the pivotal role GECs play in modulation of FGT mucosal integrity, it is likely that sensing through TLRs is involved in regulating the balance between tolerance vs defence, and modulating subsequent immunity^8^,^9^,^11^. The FGT associated lymphoid organs are part of the mucosal associated lymphoid tissue (MALT), which also includes the gastro-intestinal lymphoid tissue (GALT). Unlike the GALT, the FGT does not include M cells or organised lymphoid crypts or follicles in the sub-mucosa^11^ but contains uterine lymphoid aggregates^12^. Local immunisations at the FGT level have been shown to induce local CD4^+^, CD8^+^ CTL, IgG and IgA responses. However, mechanisms of immune induction in the FGT remain poorly understood^2^,^3^,^5^. The link with adaptive immunity mainly involves DC, the latter which through pattern recognition receptor (PRR) such as TLR sensing are also involved in maintaining a homeostatic balance between tolerance and inflammation. Cross-talk between epithelial cells and sub-mucosal DC involves immunoregulatory cytokines and lead to activation of effector cells in the lamina propria. DC are also pivotal in orchestrating innate and adaptive immune responses directly or by migrating to FGT mucosal associated draining lymphoid organs to regulate B and T lymphocyte responses^7^,^8^,^13^.

Mucosal exposure to HIV-1 in the absence of infection was documented in different cohorts across the world, including the Beninese commercial sex workers (CSWs) and evidence supports a major role for the FGT microenvironment and innate immune system in sustaining resistance against HIV-1 infection^2^,^14^,^15^. The demonstration of the presence of low-inflammatory conditions in the FGT of HIV-1 highly-exposed seronegative (HESN) Beninese and Kenyan CSWs has recently been described^16^,^17^,^18^,^19^. However, the biological impact of HIV-1 on FGT immunity and how infection could be avoided/controlled are still largely unresolved partially because studies on HIV FGT immunity remain challenging, principally due to the difficulty of recruiting participants, obtaining fresh samples, adequate numbers of viable cells, and controlling for major confounders such as sexually transmitted infections, menstrual cycle and risk behaviours.

Based on our previous studies^17^,^18^,^20^ and others^16^,^19^,^21^,^22^, we hypothesized that maintenance of low-inflammatory conditions in the FGT of HESN individuals helps to prevent excessive immune activation and lower HIV-1 target availability, likely maintaining the integrity of the mucosal barrier to protect from HIV-1 infection^2^. In the present study, we aimed to further characterize immune cells that might be involved in the production of the low-inflammatory conditions observed in the FGT of HIV-1 HESN CSWs.

## Results

### Socio-demographic characteristics of the study population

The socio-demographic characteristics of female CSWs and non-CSWs are shown in Table 1. HIV-1-infected CSWs were older than the HIV-1-uninfected (HESN) CSWs and non-CSWs. All women were practicing vaginal douching. Duration of sex work, average number of clients and condom use were similar between the HESN CSWs and HIV-1-infected CSW groups.

### Cytokines and soluble HLA-G expression levels in cervico-vaginal lavages (CVLs) from HESN CSWs, HIV-1-infected CSWs and HIV-1-uninfected non-CSW women

**Figure 1:**
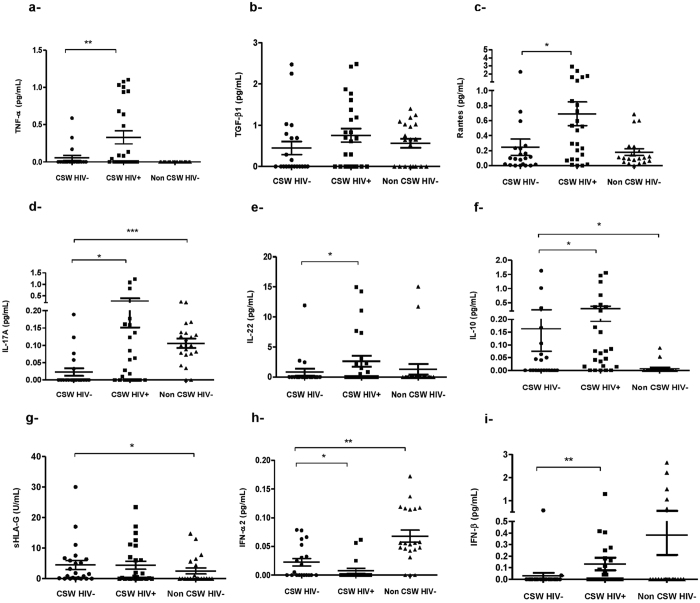
Cytokines/chemokines and sHLA-G levels in cervicovaginal lavages (CVLs) of HIV-1-uninfected CSWs, HIV-1-infected CSWs and HIV-1-uninfected non-CSW controls. Levels of TNF-α (**a**), TGF-β1 (**b**), Rantes (**c**), IL-17A (**d**), IL-22 (**e**), IL-10 (**f**), HLA-G (**g**), IFN-α2 (**h**), IFN-β (**i**) were measured in CVLs of the three study groups. Sample measurements below the Lower Detection Limit (LDL) were assigned a value of 0. Cytokine/Chemokine values are expressed in pg/mL and sHLA-G values are expressed in U/mL. P-values for the comparison between two groups were calculated with a Mann-Withney U test. Data shown are Mean ± SD. *p < 0.05, **p < 0.001 and ***p < 0.0001. CSW, commercial sex worker; HIV-1, human immunodeficiency virus type 1; TNF, tumor necrosis factor; TGF-β, transforming growth factor; Rantes, regulated on activation normal T cell expressed and secreted; IL, interleukin; IFN, interferon; and sHLA-G, soluble human leukocyte antigen-G.

Levels of TNF-α, Rantes, IL-17A, IL-10, IL-22 and IFN-β were lower in the CVLs from HESN CSWs when compared to those measured in the CVLs from HIV-1-infected CSWs (Fig. 1). Interestingly, HESN CSWs had higher CVL levels of IFN-α than HIV-1-infected CSWs (Fig. 1h). HESN CSWs had similar CVL levels of TNF-α, TGF-β, Rantes, and IL-22 (Fig. 1a–c,e), lower levels of IL-17A, IFN-α and -β (Fig. 1d,h,i), and higher levels of IL-10 and soluble HLA-G (sHLA-G) ((Fig. 1f–g) than HIV-1-uninfected non-CSW women. In agreement with previous studies^16^,^17^,^18^,^19^,^20^,^21^,^22^, these observations show that the FGT of HESN CSWs exhibit relatively low levels of pro-inflammatory cytokines in comparison to HIV-1-infected CSWs and high levels of regulatory molecules such as IL-10 and sHLA-G in comparison to HIV-1-uninfected non-CSW women.

### Distribution of cell populations in endocervical samples from HESN CSWs, HIV-1-infected CSWs and HIV-1-uninfected non-CSW women

Due to the low leukocyte yield in samples from CVLs, cells of the epithelial, myeloid and lymphoid lineages were characterized in endocervical samples following multi-colour flow cytometric analysis. The GECs (CD45^−^CK^+^) were the most abundant cell population (42%) found in the endocervical samples, followed by CD45^+^ CK^−^ leukocytes negative for the lineage markers CD3, CD19, and CD56 (41%). The latter group of cells were divided into two groups based on HLA-DR expression; myeloid (HLA-DR^+^) cells (6%) and granulocytes (HLA-DR^−^, CD66b^+^) (35%). Cells carrying markers of the lymphoid lineage CD45^+^ CK^−^CD3^+^ (T-cells) or CD19^+^ and/or CD138^+^ (B-cells, and plasmablasts and/or plasma cells) or CD56^+^ (NK-cells) represented 17% of the total endocervical cell population. The relative frequencies of these cell populations were similar between the three study groups (data not shown).

### Phenotypic characterisation of epithelial cells in endocervical samples from HESN CSWs, HIV-1-infected CSWs and HIV-1-uninfected non-CSW women

**Figure 2:**
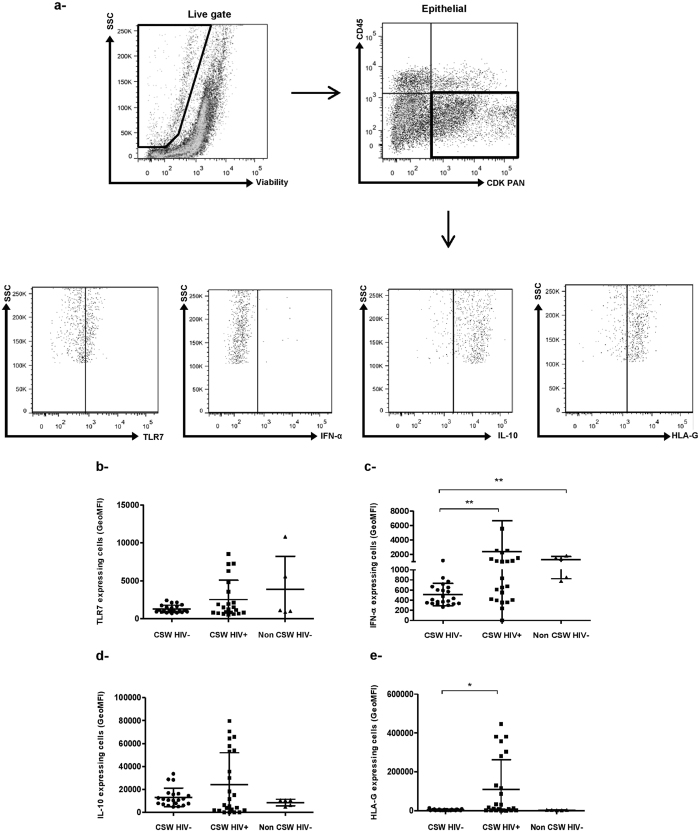
Flow-Cytometry analysis of live endocervical epithelial cells of HIV-1-uninfected CSWs, HIV-1-infected CSWs and HIV-1-uninfected non-CSW controls. (**a**) Gating strategies by flow-cytometry of an endocervical sample. Cells were first gated on live endocervical cells and then on CD45^−^CDKPAN^+^ epithelial cells. Epithelial cells from the three study groups were characterized for their intensity of expression of TLR7 (**b**), IFN-α (**c**), IL-10 (**d**) and HLA-G (**e**). Data are expressed as Geometric mean fluorescence intensity (GeoMFI). Representative FMO staining controls can be viewed in Supplementary Fig. S1. Statistical significance of differences in levels of expression (GeoMFI) was evaluated with Mann-Withney U test. *p < 0.05 and **p < 0.001. CSW, commercial sex worker; TLR, Toll-like receptor; IL, interleukin; IFN, interferon; HLA, human leukocyte antigen.

We evaluated the GECs’ expression of TLR-7, IFN-α, IL-10 and HLA-G found to be of major importance for their antiviral and immune-regulatory properties^20^,^23^,^24^,^25^,^26^,^27^. The GEC levels of expression of TLR-7, IFN-α, IL-10 and HLA-G were lower in HESN CSWs when compared to those found in HIV-1-infected CSWs, reaching significant levels for IFN-α and HLA-G (Fig. 2). Levels of TLR7 and IFN-α expression by GECs were lower in HESN when compared to those in HIV-1-uninfected non-CSWs (Fig. 2b,c), whereas similar levels of expression were found for IL-10 and HLA-G between the two groups (Fig. 2d,e).

### Characterisation of HLA-DR^+^ myeloid cells in endocervical samples from HESN CSWs, HIV-1-infected CSWs and HIV-1-uninfected non-CSW women

**Figure 3:**
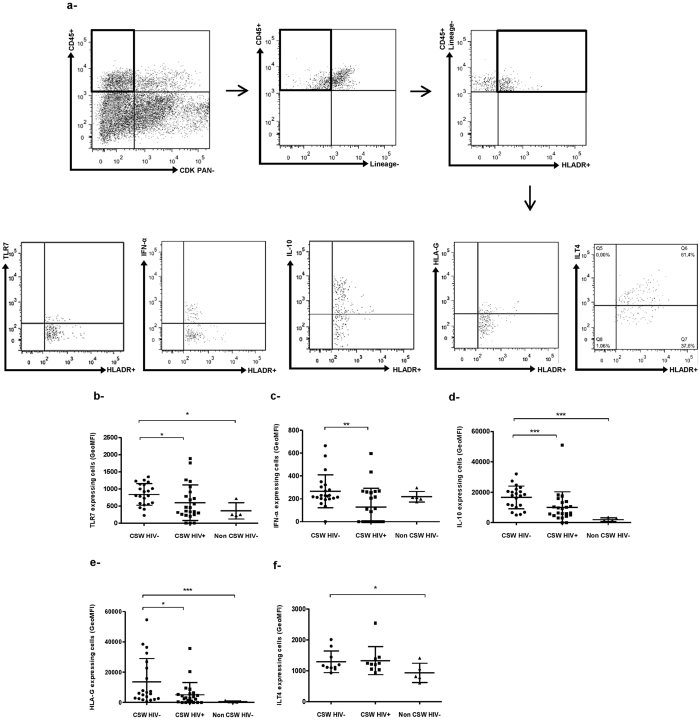
Flow-Cytometry analysis of live endocervical myeloid HLA-DR+ cells of HIV-1-uninfected CSWs, HIV-1-infected CSWs and HIV-1-uninfected non-CSW controls. (**a**) Gating strategies by flow-cytometry of an endocervical sample. Cells were first gated on live endocervical cells and then on CD45^+^ CDKPAN^−^ leukocytes. Leukocytes were gated on CD45^+^ Lineage^−^ and subsequently on HLA-DR^+^. Lineage-HLA-DR^+^ cells from the three study groups were characterized for their expression levels of TLR7 (**b**), IFN-α (**c**), IL-10 (**d**), HLA-G (**e**) and ILT-4 (**f**). Data are presented as geometric mean fluorescence intensity (GeoMFI). Representative FMO staining controls can be viewed in Supplementary Fig. S2. Statistical significance of differences in levels of expression (GeoMFI) was evaluated with Mann-Withney U test *p < 0.05, **p < 0.001 and ***p < 0.0001. CSW, commercial sex worker; TLR, Toll-like receptor; IL, interleukin; IFN, interferon; HLA, human leukocyte antigen; ILT, immunoglobulin like transcript.

The levels of expression of TLR-7, IFN-α, IL-10 and HLA-G by endocervical lineage^−^HLA-DR^+^ myeloid cells were significantly higher in HESN CSWs when compared to those observed in both HIV-1-infected CSWs and HIV-1-uninfected non-CSW women (Fig. 3b–e). Levels of immunoglobulin like transcript (ILT)-4 expression by myeloid cells were similar for the two CSW groups (Fig. 3f), but expression was significantly higher in myeloid cells from the HESN CSWs when compared to those from HIV-1-uninfected non-CSW women (Fig. 3f).

**Figure 4:**
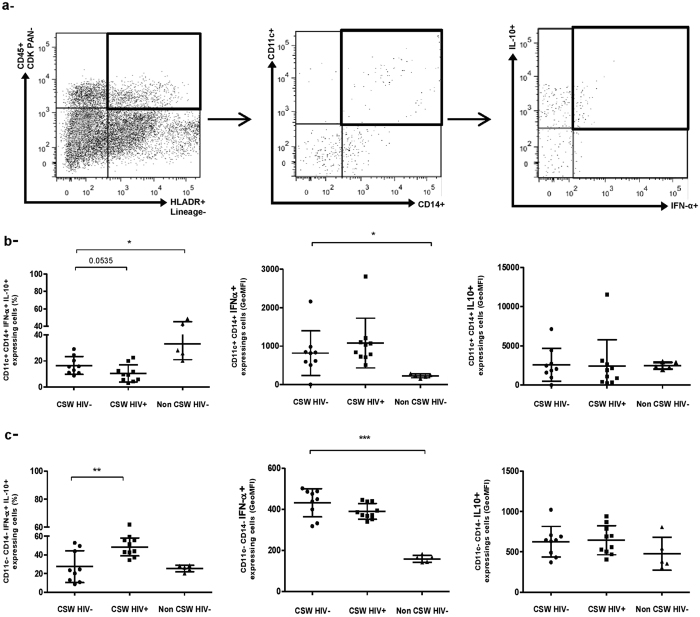
Flow-Cytometry analysis of live endocervical myeloid HLA-DR + CD11c + CD14+ and HLA-DR + CD11c-CD14- cells of HIV-1-uninfected CSWs, HIV-1-infected CSWs and HIV-1-uninfected non-CSW controls. (**a**) Gating strategies by flow-cytometry of an endocervical sample. Cells were first gated on live endocervical cells and then on CD45^+^ CDKPAN^−^ leukocytes. Leukocytes were gated on CD45^+^ Lineage^−^ and subsequently on HLA-DR^+^. Lineage^−^HLA-DR^+^ cells were then gated based on their expression of CD11c and/or CD14 and subsequently IFN-α and IL-10. (**b**) The frequencies (%) of CD11c^+^ CD14^+^ IFN-α^+^ IL-10^+^ cells (left panel), and geometric mean fluorescence intensity (GeoMFI) of their IFN-α expression levels (middle panel) and IL-10 (right panel) expression levels are shown for the three study groups. (**c**) The frequencies (%) of CD11c^−^CD14^−^IFN-α^+^ IL-10^+^ cells (left panel), and GeoMFI of their IFN-α expression levels (middle panel) and IL-10 (right panel) expression levels are shown for the three study groups. Statistical significance of differences in the relative frequencies (%) and levels of expression (GeoMFI) were evaluated with Mann-Withney U test when statistical no-parametric and with Unpaired T test when statistical parametric. *p < 0.05, **p < 0.001 and ***p < 0.0001. CSW, commercial sex worker; IL, interleukin; IFN, interferon.

Further characterization of the endocervical myeloid lineage^−^HLA-DR^+^ cells revealed a population expressing CD11c and CD14 as well as both IFN-α and IL-10. The relative frequencies of these CD11c^+^ CD14^+^ IFNα^+^ IL-10^+^ cells in HESN CSWs were higher than those in HIV-1-infected CSWs but lower than in those from HIV-1-uninfected non-CSW women (Fig. 4b left panel). The majority of this cell subset expressed CD103 (80%) and HLA-G (87%), and a relatively large proportion also expressed CD1a (66%) and ILT-4 (55%). Intensity of expression of IFN-α and IL-10 by these cells were similar between the three groups (Fig. 4b middle and right panels), except for lower levels of IFN-α expression in the HIV-1-uninfected non-CSW women (Fig. 4b middle panel). We also found a population of myeloid lineage^−^HLA-DR^+^ which were negative for CD11c and CD14 but positive for both IFN-α and IL-10. In contrast to the CD11c^+^ CD14^+^ IFNα^+^ IL-10^+^ subset, a smaller proportion of the CD11c^−^CD14^−^IFNα^+^ IL-10^+^ cells were positive for CD103 (30%), HLA-G (40%) and ILT-4 (36%), whereas the proportion of cells expressing CD1a (72%) was similar. The relative frequencies of the CD11c^−^CD14^−^IFNα^+^ IL-10^+^ cell subset were lower in the HESN CSWs when compared to the HIV-1-infected CSWs (Fig. 4c left panel). Intensity of expression for IFNα and IL-10 by these cells were similar between the three groups (Fig. 4c middle and right panels), except for lower levels of IFN-α expression in the HIV-1-uninfected non-CSW women (Fig. 4c middle panel).

### Characterisation of CD4^+^ regulatory T-cell populations in endocervical samples from HESN CSWs, HIV-1-infected CSWs and HIV-1-uninfected non-CSW women

**Figure 5:**
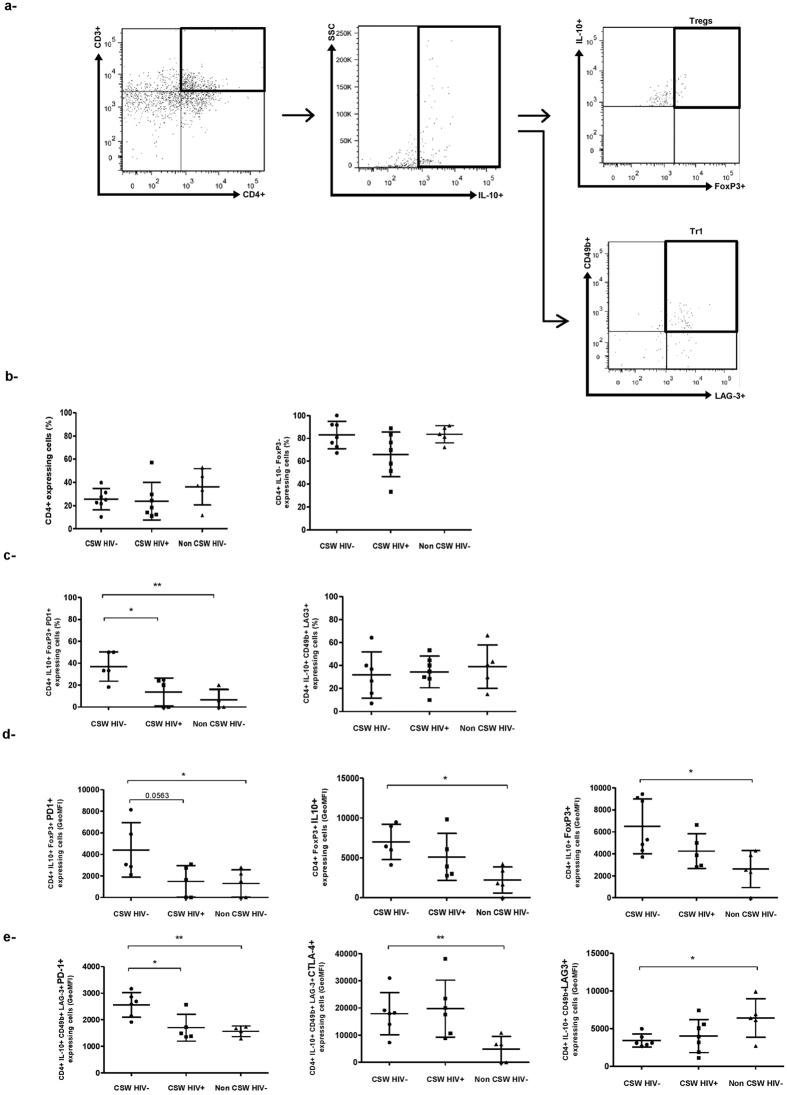
Flow-Cytometry analysis of live endocervical CD4^+^ T-cells of HIV-1-uninfected CSWs, HIV-1-infected CSWs and HIV-1-uninfected non-CSW controls. (**a**) Gating strategies by flow-cytometry of an endocervical sample. Cells were first gated on live endocervical cells and then on CD3^+^ CD4^+^ T-cells. CD4^+^ T-cells were selected based on expression of IL-10^+^ FoxP3^+^ (Treg) and IL-10^+^ CD49b^+^ LAG-3^+^ (Tr1). The frequencies (%) of (**b**) total CD4^+^ T-cells (left panel) and CD4^+^ IL-10^−^FoxP3^−^ T-cells (right panel), and (**c**) CD4^+^ IL-10^+^ FoxP3^+^ PD-1^+^ Treg (left panel) and CD4^+^ IL-10^+^ CD49b^+^ LAG-3^+^ Tr1 cells (right panel) are shown for the three study groups. The geometric mean fluorescence intensity (GeoMFI) of (**d**) PD-1 (right panel), IL-10 (middle panel) and FoxP3 (right panel) is presented for CD4^+^ IL-10^+^ FoxP3^+^ Tregs, and (**e**) PD-1 (right panel), CTLA-4 (middle panel) and LAG-3 (right panel) is presented for CD4^+^ IL-10^+^ CD49b^+^ LAG-3^+^ Tr1 cells. Representative FMO staining controls can be viewed in Supplementary Fig. S3. Statistical significance of differences in the relative frequencies (%) and levels of expression (GeoMFI) were evaluated with Unpaired T test. *p < 0.05 and **p < 0.001. CSW, commercial sex worker; IL, interleukin; LAP, latency associated peptide; FoxP3, forkhead box P3; PD-1, Programmed cell death protein 1; LAG-3, Lymphocyte activation gene-3.

We found no significant difference in the percentages of total endocervical CD4^+^ T-cells or CD4^+^ IL-10^−^FoxP3^−^ likely “effector” T-cells between the three study groups (Fig. 5b left and right panels). Given our finding that genital myeloid cells from HESN CSWs presented a more “tolerogenic/regulatory” profile, we characterised the CD4^+^ T-cell regulatory profile. We identified two distinctive CD4^+^ T-cell regulatory phenotypes in endocervical samples of the three study groups, namely CD4^+^ IL-10^+^ FoxP3^+^ T regulatory cells (Tregs) and CD4^+^ IL-10^+^ CD49b^+^ LAG3^+^ type 1 regulatory T cells (Tr1)^28^. We also found expression of the molecules programed cell death protein (PD)-1, TGF-β latency associated peptide (LAP), inducible costimulatory (ICOS), lymphocyte-activation gene (LAG)-3 and cytotoxic T-lymphocyte associated protein (CTLA)-4 on a great percentage of cells within these two cellular types. The relative frequencies of Tregs expressing PD-1 were significantly increased in the HESN CSWs when compared to both HIV-1-infected CSWs and HIV-1-uninfected non-CSW women (Fig. 5c left panel). The frequencies of Tr1 were similar between the three groups (Fig. 5c right panel). Interestingly, the intensity of expression of PD-1 was greater in both Tregs and Tr1 from HESN CSWs when compared to the two other groups (Fig. 5d,e left panels). Moreover, Tregs of HESN CSWs had higher IL-10 and FoxP3 expression levels than those from both HIV-1-infected CSWs and HIV-1-uninfected non-CSWs, reaching significant differences with the latter group (Fig. 5d middle and right panels). Levels of CTLA-4 and LAG3 expression in Tr1 from HESN CSWs were similar to those in HIV-1-infected CSWs but different than those in HIV-1-uninfected non-CSW women (Fig. 5e middle and left panels).

## Discussion

We and others have shown that natural immunity to HIV-1 in HESN CSWs is associated with low genital inflammatory conditions^16^,^17^,^18^,^19^,^20^,^21^,^22^. To further our understanding of the mechanisms that orchestrate this low inflammatory profile and confer protection against HIV-1, we have characterized and compared inflammatory vs “tolerogenic/regulatory” cytokines as well as the phenotype of genital immune cells and their “tolerogenic/regulatory” profile in the FGT of CSWs from Benin.

As expected, HESN CSWs had lower levels of pro-inflammatory cytokines in their genital fluids than did the HIV-1-infected CSWs. Interestingly, relatively higher levels of IFN-α were found in HESN CSWs, which could be critical to sustain immune homeostasis, antiviral activity and restriction factors in cells at the portal of entry for the virus. Indeed, following viral encounter/infection, the induced IFNs can upregulate a myriad of IFN-stimulated genes (ISGs), which have been shown to interfere with multiple viruses at various life cycle stages^27^,^29^. Moreover, a recent study demonstrated that blockade of the IFN-I receptor caused reduced antiviral gene expression, increased SIV reservoir size and accelerated CD4^+^ T cell depletion with progression to AIDS^30^. The elevated IFN levels observed in the FGT of HIV-1-uninfected non-CSWs also suggest that these African women might be exposed to microbial factors or have inflammatory/infectious conditions other than HIV favouring IFN production.

The relatively high levels of IL-22 and IL-17A in CVLs of HIV-1-infected CSWs may possibly reflect Th17 activity and an attempt from the infected host to preserve mucosal integrity^31^,^32^. IL-22 in conjunction with IL-17A or IL-17F synergistically induced the expression of β-defensin 2 and other antimicrobial peptides (S100A9, S100A7 and S100A8)^32^. However, sustained Th17 activity may lead to barrier impairment, increasing epithelium permeability and allowing for microbial translocation and chronic inflammation/activation^33^. On the other hand, the relatively high levels of IL-22 and IL-17A found in the CVLs from HIV-1-infeted CSWs may be produced by cell types other than Th17 such as innate lymphoid cells, NK, macrophages and neutrophils, therefore additional experiments are warranted to directly assess Th17 activity and mucosal integrity in the genital mucosa of CSWs. GECs from HESN CSWs expressed low levels of expression of TLR7, IFN-α, IL-10 and HLA-G when compared to those observed in HIV-1-infected CSWs. As to whether the discrepancy between IFN-α levels measured in the CVLs and those expressed by GECs of HESN and HIV-1-infected CSWs is linked to differential mechanistic and/or kinetics of a production/release/consumption loop remain to be established.

However, in contrast to that observed for GECs, endocervical myeloid HLA-DR^+^ cells from HESN CSWs expressed higher levels of IFN-α as well as TLR-7, IL-10 and HLA-G when compared to both HIV-1-infected CSWs and HIV-1-uninfected non CSWs. This is consistent with the elevated IFN-α and IL-10 levels we measured in the CVLs of HESN CSWs possibly to promote a potent antiviral and yet at the same time immunoregulatory profile. IL-10 levels are often elevated in the context of HIV, as reported here in the CVLs from HIV-1-infected CSWs, but the overall outcome of excessive IL-10 may well be to sustain chronic activation and dysregulation, and may lead to imbalanced Treg/Teffector ratios^34^,^35^ associated with HIV disease progression^2^,^3^. Furthermore, high level of regulatory activity may impede on viral eradication^36^. In contrast, a more modest elevation of IL-10, such as observed for HESN CSWs, may be beneficial and promote an immunoregulatory microenvironment^37^. Although, additional experiments are needed to directly assess the impact of IL-10 on the production and functionality of Tregs observed in the mucosal samples of HESNs, several studies support the role of IL-10 in promoting immunoregulatory responses. Both IFN-α and IL-10 are involved in differentiation of long-lasting antigen-specific T-cell anergy and Tr1^38^,^39^. *In vitro* studies have demonstrated that monocyte derived DCs treated with IL-10 and/or IFN-α were rendered “tolerogenic” and upregulated the inhibitory receptors ILT-3 and ILT-4, which promoted their capacity to induce Tr1^24^,^40^. IL-10 is one of the key cytokines inducing HLA-G expression on myeloid cells^41^. The engagement of the inhibitory molecules ILT-2, ILT-3 and ILT-4 on myeloid cells by HLA-G prevents the up-regulation of costimulatory molecules, inhibits maturation and allows them to promote the differentiation of CD4^+^ Tregs^26^,^42^. It has been reported that, in addition to its membrane-bound form, sHLA-G also plays a role in promoting the induction of Tregs^43^. Amodio and colleagues identified a subset of “tolerogenic” DCs, named DC-10 that secrete high amounts of IL-10, express high levels of HLA-G and ILT-4 and bind CD4^+^ HLA-G^+^ T-cells at the fetal maternal interface, where they may contribute to tolerance^23^. Furthermore, Gregori and colleagues have demonstrated that these DC-10 can induce Tr1 via an IL-10–dependent ILT4/HLA-G pathway^25^. Interestingly, we found a subset of myeloid HLA-DR^+^ CD11c^+^ CD14^+^ “DC” bearing IFN-α and IL-10, which relative percentage is increased in endocervical samples of HESN CSWs. The fact that we found a significant increase in IFN-α expressing cells to be associated with phenotypically distinct myeloid HLA-DR^+^ cells in endocervical samples of HESN CSWs supports the notion that production of IFN-α may be tightly regulated and restricted in these individuals. These myeloid HLA-DR^+^ CD11c^+^ CD14^+^ IFN-α^+^ IL-10^+^ cells also expressed high levels of CD103, which could correspond to their function as mucosal sentinels poised to respond to and translate microbial antigens to cells of the innate and adaptive immune response, as has been reported for similar populations in other mucosal sites^44^. Also, murine CD103^+^ DCs^45^, and human CD103^+^ DCs found throughout the gut lamina propria are thought to be more effective at promoting Treg responses and therefore play a central role in maintaining tolerance and tissue homeostasis^13^. Interestingly, the majority of the myeloid CD11c^+^ CD14^+^ IFN-α^+^ IL-10^+^ cells also expressed HLA-G and ILT-4, as do DC-10^23^,^25^. It is thus possible that the CD11c^+^ CD14^+^ IFN-α^+^ IL-10^+^ myeloid population mostly bearing CD103 and HLA-G and found to be relatively increased in endocervical samples of HESN CSWs play a similar role in the FGT, which would be consistent with the increased frequencies of endocervical Tregs we found for these individuals. This is also in agreement with previous findings showing elevated frequencies of Tregs in the blood of HESN CSWs from Kenya^46^. The higher expression levels of PD-1 we report for endocervical Tregs and Tr1 of HESNs could reflect T-cell exhaustion, however we prefer the view by which these cells are in an homeostatic activated status, possibly regulating cytotoxic T lymphocyte (CTL) or DC activities via PD-1L^47^,^48^, which likely confers an advantage to these individuals. Furthermore, endocervical Tregs and Tr1 cells from HESN also expressed higher levels of IL-10 and CTLA-4, respectively, which again reflect their regulatory activity^47^.

Inversely, the myeloid HLA-DR^+^ CD11c^−^CD14^−^IFN-α^+^ IL-10^+^ subset, which frequencies are significantly increased in the FGT of HIV-1-infected CSWs, expressed lower levels of CD103 and HLA-G. Interestingly, in contrast to CD103^+^ DCs, CD103^−^ DCs have a more immunogenic phenotype in both the steady state^49^ and in an inflammatory context^50^. Moreover, CD103^−^ intestinal mouse DCs have been shown to induce differentiation of IFN-γ and IL-17-producing effector T cells^34^. Therefore, the CD103^−^ myeloid cell subset found to be elevated in the FGT of HIV-1-infected CSWs could have a more “activated” phenotype than the CD103^+^ myeloid subset found in HESN CSWs. It is thus possible that the CD103^−^ myeloid cell subset found in HIV-1-infected CSWs may contribute to the inflammatory conditions observed in the genital mucosa of these women resulting in increased susceptibility to HIV-1 infection and disease progression/perpetuation at the initial site of exposure.

Because of the cross-sectional design, the present study cannot address whether the myeloid subsets and Tregs found to be increased in the FGT of HESN CSWs, have a protective role against HIV infection. Moreover, the control group HIV-uninfected non-CSWs differ from the HESN study group by both HESN status and exposure to sex work. Therefore it is not possible to determine if the differences observed between these groups are due to regular exposure to sex antigens and semen of clients or if it is due to the HESN phenotype. A longitudinal study to compare samples before and after seroconversion and further phenotypic and functional characterisations are required to confirm the protective role, the exact nature of the myeloid and regulatory T-cells, and whether they represent different populations or stages of differentiation remain to be established. Nevertheless the findings reported herein support the hypothesis that natural immunity/resistance to HIV-1 infection may be orchestrated by specific mucosal tolerogenic/regulatory myeloid cell populations, which promote Tregs and induce a potent antiviral but regulated immune response to prevent excessive immune activation, lower HIV-1 target availability, and maintain the integrity of the mucosal barrier.

## Methods

### Study population

Female CSWs were recruited through a dedicated sex worker clinic in Cotonou, Benin. Non-CSW control women at low risk for exposure were enrolled from a general health clinic in Cotonou. Women were invited to participate in the study as they attended clinics. Women were excluded from the study if, they were less than 18 years old, menstruating or pregnant. At enrolment, participants were asked to answer a questionnaire about demographic information, sexual behaviour, duration of sex work, number of sex partners, condom use, vaginal douching practices, and reproductive history. Each participant underwent a genital examination by a physician. Vaginal specimens were obtained for diagnosis of candidiasis, trichomoniasis and bacterial vaginosis by microscopic examination and HSV infection by PCR. Endocervical swabs were obtained to test for *Neisseria gonorrhoeae* and *Chlamydia trachomatis* infection using BD ProbeTec ET system (Strand Displacement Assay, Becton Dickinson, Heidelberg, Germany). Peripheral blood was taken for HIV, syphilis, HSV and progesterone testing by immunoassays. HIV-1 positivity was defined by the presence of HIV-1 specific IgG tested with Vironostika HIV Uni-Form II Ag/Ab (Organon Teknika, Boxtel, The Netherlands). Non-reactive samples were considered HIV seronegative, whereas reactive samples were tested with Genie II HIV-1/HIV-2 (Bio-Rad, Hercules, CA). Genie II dually reactive samples (to HIV-1 and HIV-2) and discordant samples (Vironostika reactive/Genie II non-reactive) were further tested by INNO-LIA HIV I/II Score (Innogenetics NV, Technologiepark 6, Gent, Belgium). HSV infection and shedding was determined by testing for HSV-specific antibodies in the serum and for the presence of HSV in the CVLs of the women by PCR assay. In the present study we selected genital samples from 22 HIV-1-uninfected CSWs or HESN, 24 treatment-naïve HIV-1-infected CSWs and 13 HIV-1-uninfected non-CSW control women. For the phenotype characterisation of GEC, myeloid and CD4^+^ T cells, endocervical samples from 9 or 7 HIV-1-uninfected CSWs, 10 or 7 treatment-naïve HIV-1-infected CSWs and 5 HIV-1-uninfected non-CSW women were available, respectively. The three study groups were all in the follicular phase of their menstrual cycle as determined by progesterone levels, not taking oral contraception, and had no co-infection, bacterial vaginosis, trichomoniasis or candidiasis. The average blood CD4^+^ T-cells count for HIV-1-infected CSWs was 500 cells/mm3.

### Ethics statement

Written informed consent was obtained from all subjects who participated in the study. The methods reported in this paper were performed in accordance with the relevant guidelines and regulations and all experimental protocols were approved by the Comité National Provisoire d’Éthique de la Recherche en Santé in Cotonou and the Centre Hospitalier de l’Université de Montréal (CHUM) Research Ethics Committees.

### Cervico-vaginal lavage (CVL) sample collection and preparation

CVL samples were obtained from all study participants by a physician, using a 10-ml syringe filled with sterile 1x phosphate-buffered solution (PBS) and aimed directly into the cervical os. CVL fluids were then collected, transferred immediately into 20 ml of RPMI-1640, kept on ice, and processed within 1 hour. CVL samples were centrifuged at 1500 rpm for 10 min and supernatants were concentrated on a 3 KDa Amicon membrane and were stored at −80 °C until shipped on dry ice to Montreal, Canada. CVL cells were cryopreserved in liquid nitrogen until shipped in transport tanks to Montreal, Canada.

### Endocervical cell sample collection and preparation

Endocervical cells were collected using a cytobrush under speculum examination by inserting the cytobrush into the cervix, rotating 360° and immediately placing in 5 mL of RPMI. Cytobrush samples with visible blood contamination were excluded from further analysis. Samples were kept on ice and processed within 1 hour. The cytobrush was vortexed, cells were flushed out of the brush, suspended in freezing medium (90% heat inactivated fetal bovine serum (hi-FBS), 10% DMSO) and cryopreserved in liquid nitrogen until shipped in transport tanks to Montreal, Canada.

### Cytokines measurement

Cytokines were measured in CVLs using the ProcartaPlex immunoassay (Affimetrix/eBioscience, San Diego, CA, USA), which allows simultaneous detection of IFN-β, IL-10, IL-17A, IL-22, and TNF-α, IFN-α was quantified by Bio-Plex cytokine/chemokine assay (Bio-Rad, Hercules CA, USA) and TGF-β was quantified by Milliplex (Millipore, Billerica, MA, USA). Analysis was performed on a Luminex^®^ 200 System (Luminex Corporation, Austin, TX, USA). The final concentration for a given cytokine in the CVL sample was determined as follows: concentration obtained with the Luminex analyser (pg ml^−1^)/(CVL concentration factor). The concentration factor was calculated as follows: initial volume/final volume (after concentration). Samples below the LDL were assigned a value of 0 pg/ml. sHLA-G levels were measured using the Human sHLA-G Immunoassay kit (Alexis Biochemicals, San Diego, CA, USA), which allows simultaneous detection of HLA-G1 and -G5 soluble proteins without discrimination. The final concentration of sHLAG in the CVL sample was determined as follows: concentration obtained with the sHLA-G Elisa assay (units per ml)/(CVL concentration factor).

### Flow-Cytometry analyses

Endocervical cells from cytobrush samples were thawed, washed and processed for flow-cytometry analysis. Briefly, a maximum of 2 × 10^5^ endocervical cells per well were used for staining. Live/dead exclusion was performed using Aqua-LIVE/DEAD Fixable Stain (Invitrogen Life technologies, Eugene, OR, USA). Non-specific binding sites were blocked using fluorescence-activated cell sorting (FACS) buffer (1x PBS, 2% heat inactivated (hi)-FBS, and 0.1% sodium azide) supplemented with 20% hi-FBS and 10 ug mouse and/or rat IgG (Sigma-Aldrich, St-Louis, MO, USA). The following conjugated mouse or rat anti-human monoclonal antibodies were used: anti-CD3-e fluor 605 NC, anti-CD138-Pe, anti-CD158a-Pe Cy7, anti-CD3/anti-CD19/anti-CD56-Per CP e fluor 710, anti-CD14-e fluor 605 NC, anti-CD11c-Pe, anti-HLA-DR-APC 780, anti-BDCA2-Pe Cy7, and anti-CD11c Pe Cy7, anti-CD103-FITC, anti-CD8-APC 780, anti-LAP-Pe, and anti-FOXP3-488 (eBioscience, San Diego, CA, USA); anti-CD66b-FITC, anti-CD1a-a fluor 700, and anti-CD207-Pe, anti-ILT4-APC, anti-CD56-Brilliant Violet 510, and rat anti-IL-10-Pe Cy7 (BioLegend, San Diego, CA, USA); anti CD19-FITC, anti-CD45-V450, anti-CD209-Pe CF594, anti-HLA-G-biotin/Streptavidin-Pe CF594, anti-IFN-α2b-Pe, anti-TLR7-FITC, anti-CD11c-Pe CF594, HLA-G-Per CP Cy5.5, anti-CD138-BV510, anti-CD4-a fluor 700, and anti-CCR5 (CD 195)-Pe CF504 (BD-Biosciences); anti-Pan Cytokeratin-APC (EXBIO Praha Czech Republic); anti-CD19-Pacific Green (Life Technologies); anti-CD45RA- BV711 (BioLegend, San Diego, CA, USA); anti-LAG-3- Pe, (R&D Systems, Minneapolis, USA); anti-CD3- eVolve 605, anti-ICOS- APC (eBioscience, San Diego, CA, USA); anti-CD45- BUV395, anti-CD49- BV786, anti-CTLA-4- BV421, anti-PD-1- PerCP Cy 5.5, anti-CD8-APC/H7, anti-CD4- a fluor 700, and anti-LAP- PeCF 594 (BD-Biosciences). Intracellular labelling was performed using the Cytofix/Cytoperm Fixation/Permeabilization kit and perm/wash buffer (BD-Biosciences). Intracellular non-specific binding sites were blocked using perm/wash buffer containing 20% hi-FBS, 50% rat serum and 20 ug mouse IgG. Cells were kept at 40 C in 1.25% paraformaldehyde for 18 hours prior to analysis. Data acquisition of 5 × 10^4^ events per sample was performed with LSRFortessa (BD-Biosciences), and analysis was done with FlowJo7.6.3 software (TreeStar, Ashland, OR, USA). All stainings were compared to that of fluorescence minus one (FMO) values (Supplementary Fig. 1) and isotype controls. Anti-mouse Ig(κ) and Anti-Rat Ig(κ) Compbeads (BD-Biosciences) were used to optimize fluorescence compensation settings. CS&T beads (BD) were routinely used to calibrate the LSRFortessa to exclude the possibility of instrument-related fluorescence intensity changes over time, and we verified consistency prior to each data acquisition session using application settings based on Rainbow beads (BD).

### Statistical analyses

Data from HESN CSWs were compared separately with those of HIV-1-infected CSWs and HIV-1-uninfected non-CSWs. The statistical significance of difference between groups was determined by Fisher’s exact test for categorical variables and unpaired Student’s T-test or one-way ANOVA analysis for variance when continuous variables were normally distributed or by Mann-Whitney U test otherwise. The D’Agostino-Pearson normality test was used to determine whether the values were sampled from a Gaussian distribution. Analyses were performed using GraphPad Prism 5.00 for Windows (GraphPad Software, San Diego, California, USA).

## Additional Information

**How to cite this article**: Thibodeau, V. *et al*. Highly-Exposed HIV-1 seronegative Female Commercial Sex Workers sustain in their genital mucosa increased frequencies of tolerogenic myeloid and regulatory T-cells. *Sci. Rep.* **7**, 43857; doi: 10.1038/srep43857 (2017).

**Publisher's note:** Springer Nature remains neutral with regard to jurisdictional claims in published maps and institutional affiliations.

## Supplementary Material

Supplementary Figures

## Figures and Tables

**Table 1:** Distribution of demographic and sexual behavior characteristics in HIV-1-uninfected CSWs, HIV-1-infected CSWs, HIV-1-uninfected non-CSW women.

	HIV-1-uninfected CSWs	HIV-1-infected CSWs	HIV-1-uninfected non-CSWs	p-value^a^
	N = 22	N = 24	N = 13	
Age, mean (SD), years	37 (3)	44 (8)	34 (7)	0.001
Duration of sex work, mean (SD), years	5 (1)	6 (1)	NA	NS
Number of client past week, mean (SD)	15 (10)	23 (22)	NA	NS
Condom always used with clients past week	19 (86%)	21 (88%)	NA	NS
HSV positive serology	19/22 (86%)	22/24 (92%)	8/13 (62%)	NS
Vaginal douching	22 (100%)	24 (100%)	13 (100%)	NS

CSW, commercial sex workers; HIV, human immunodeficiency virus; HSV, herpes virus; N, number of participants; NA, non-applicable; NS, non-significant; SD, standard deviation.

^a^p-value for the comparison across all groups were calculated with one-way ANOVA analysis for variance of the age; Unpaired T- test for the duration of sex work and number of clients; Fisher’s exact test for condom use, vaginal douching and HSV sero-positivity.

HIV prevalence among CSWs and male clients in Cotonou at the time of recruitment was 31% and 8.8%, respectively. The Cohort HIV seroincidence is 3.1%.
