# Supplementary material for: Highly-Exposed HIV-1 seronegative Female Commercial Sex Workers sustain in their genital mucosa increased frequencies of tolerogenic myeloid and regulatory T-cells
Source: Sci Rep. 2017 Mar 6;7:43857. doi: 10.1038/srep43857 (PMC5338327; doi:10.1038/srep43857)
Supplement: Supplementary Figures [file srep43857-s1.pdf]

Supplementary Figures

Highly-Exposed HIV-1 seronegative Female Commercial Sex Workers sustain in their genital mucosa increased frequencies of tolerogenic myeloid and regulatory T-cells

V. Thibodeau[1,2], L. Fourcade[1,2], A.-C. Labbé[2], M. Alary[3,4], F. Guédou[5], J. Poudrier[1,2], M. Roger[1,2]

[1] Laboratoire d'immunogénétique, Centre de Recherche du Centre Hospitalier de l'Université de Montréal (CRCHUM), Montréal, Canada, [2]Département de Microbiologie, Infectiologie et Immunologie de l'Université de Montréal, Montréal, Canada, [3]Centre de recherche du CHU de Québec, Québec, Canada, [4]Département de médecine sociale et préventive, Université Laval, Québec, [5]Dispensaire des IST, Cotonou, Benin.

Figure S1

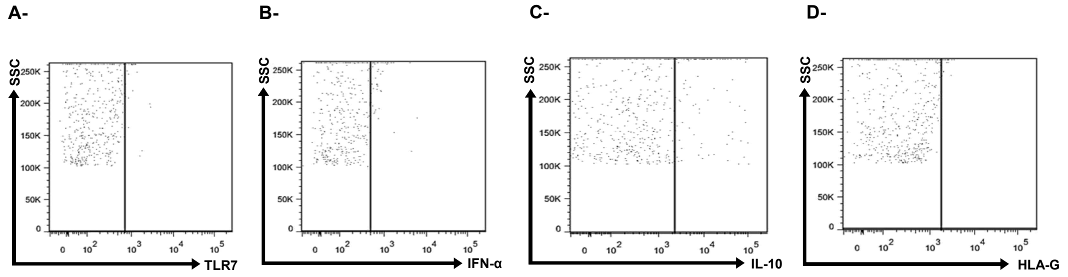

Supplementary Figure S1. Representative fluorescence minus one (FMO) staining controls for Figure 2. FMO of toll-like receptor (TLR)-7 (A), FMO of interferon (IFN)-α (B), and FMO of interleukin (IL)-10 (C), FMO of human leukocyte antigen (HLA)-G (D) staining on gated live endocervical epithelial cells .

Figure S2

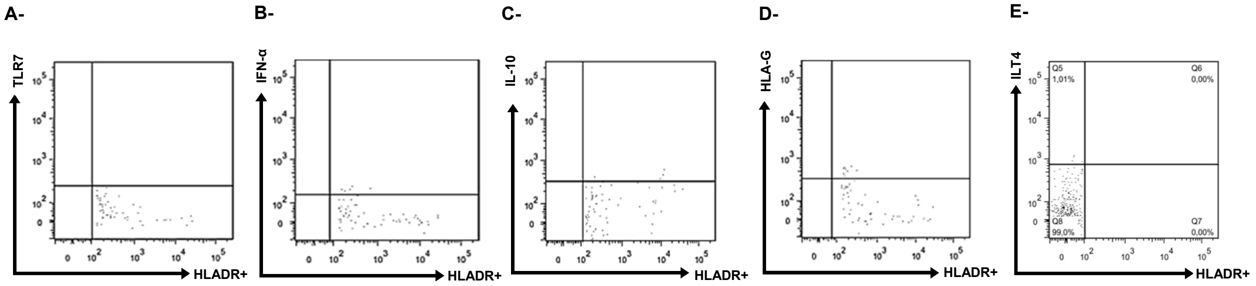

Supplementary Figure S2. Representative fluorescence minus one (FMO) staining controls for Figure 3. FMO of TLR7 (A), FMO of IFN-α (B), FMO of IL-10 (C), FMO of HLA-G (D), and FMO of immunoglobulin like transcript (ILT)-4 (E) on gated live endocervical lineage$^-$ HLA-DR$^+$ cells.

Figure S3

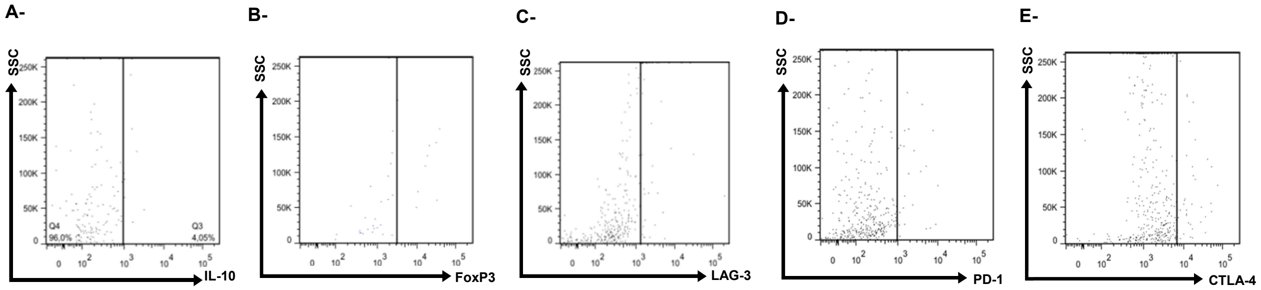

Supplementary Figure S3. Representative fluorescence minus one (FMO) staining controls for Figure 5. FMO of IL-10 (A), FMO of forkhead box P3 (FoxP3) (B), FMO of lymphocyte activation gene (LAG)-3 (C), FMO of programmed cell death protein (PD)-1 (D), and FMO of cytotoxic T-lymphocyte associated protein (CTLA)-4 (E) on gated live endocervical CD3$^+$CD4$^+$ T-cells
